# Computational Insights into Glucose Tolerance and Stimulation in a Family 1 β‑glucosidase

**DOI:** 10.1021/acs.jcim.5c00922

**Published:** 2025-07-01

**Authors:** Artur H. S. Dias, Munir S. Skaf, Rodrigo L. Silveira

**Affiliations:** † Institute of Chemistry, 28125 Federal University of Rio de Janeiro, Av. Athos da Silveira Ramos 149, 21941-909, Rio de Janeiro, RJ, Brazil; ‡ Institute of Chemistry, University of Campinas, Rua Monteiro Lobato 270, 13083-862, Campinas, Sao Paulo, Brazil; § Center for Computing in Engineering and Sciences, University of Campinas, Rua Monteiro Lobato 270, 13083-862, Campinas, Sao Paulo, Brazil

## Abstract

β-Glucosidases catalyze the hydrolysis of cellobiose to glucose during lignocellulosic biomass depolymerization. A significant limitation of many β-glucosidases is product inhibition by glucose, leading to reduced conversion efficiency. However, certain β-glucosidases exhibit tolerance or even stimulation by glucose. The mechanisms underlying this remarkable feature remain poorly elucidated. Here, we employ molecular dynamics simulations to investigate the molecular basis of glucose tolerance and stimulation within the family 1 β-glucosidase from *Humicola insolens* (*Hi*Bgl). Potential of mean force calculations reveal a substantial difference in binding free energies between cellobiose (−12.5 kcal/mol) and glucose (−4.3 kcal/mol) at the *Hi*Bgl active site, indicating that the glucose product is a considerably weaker ligand than the cellobiose substrate. These findings are consistent with our observations that *Hi*Bgl undergoes conformational changes in its substrate binding site, specifically involving the Trp349 side chain, in the presence of glucose, potentially facilitating glucose expulsion and mitigating product inhibition. Simulations of *Hi*Bgl solvated in a 200 mM aqueous glucose environment show that glucose molecules from the bulk solution are capable of penetrating and widening the substrate binding pocket, forming direct interactions with cellobiose in the active site, which may contribute to catalytic stimulation. Additionally, we identify seven distinct secondary glucose binding sites located on the *Hi*Bgl surface, spatially distant from the active site, implying a potential role in allosteric regulation. Finally, we demonstrate that glucose at subsite +1 can adopt multiple orientations relative to glucose at subsite −1, a prerequisite for transglycosylation reactions in *Hi*Bgl. Our findings elucidate the molecular mechanisms governing *Hi*Bgl’s glucose tolerance and stimulation, thereby enabling the design of site-directed mutagenesis experiments to improve enzyme efficiency for industrial applications, particularly in biofuel production and oligosaccharide synthesis.

## Graphical abstract

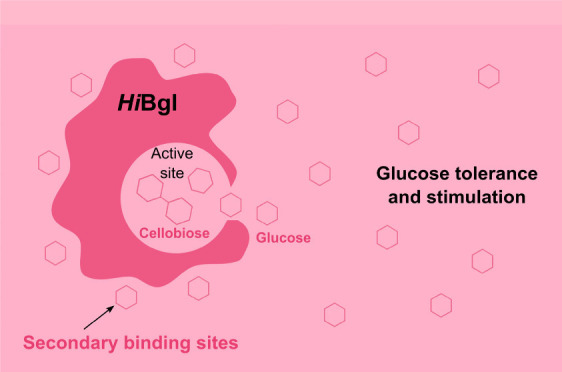

## Introduction

Global warming has become a matter of utmost concern for modern society. As human emissions of greenhouse gases and consumption of fossil fuels continue to rise, climate change effects are being felt all over the globe at ever-increasing severity.^1^ Furthermore, projected depletion of fossil fuel reserves within the coming decades underscores the unsustainable nature of our current fossil fuel-dependent economy. Consequently, the development of alternative energy sources has become a critical area of research, offering the potential to reduce reliance on finite nonrenewable hydrocarbon resources, mitigate atmospheric pollutant emissions, and foster environmentally sustainable economic sectors.^2^

In the face of such challenges, biofuels have rapidly emerged as a promising means toward a greener industry, since they are renewable and have the potential to replace oil-based fuels.^3^ Renewable biofuels are obtained from the enzymatic biotransformation of plant crops and their bagasse. Fermentable sugars are either extracted from the plant body (e.g., sugar cane extracts) or obtained from breaking down cellulose chains that make up the lignocellulosic biomass.^4^ The latter approach, also known as second-generation ethanol, is not only a viable renewable strategy, but also a means for reducing the amount of agricultural waste, as the plant bagasse can be turned into value-added biofuel and other products instead of simply being discarded.^5^

The breakdown of cellulose chains in plant biomass is achieved most efficiently through the use of enzymatic cocktails, composed mainly of endoglucanases, cellobiohydrolases, β-glucosidases, lytic polysaccharide monooxygenases (LPMOs),^6,7^ and the most recently discovered cellulose oxidative cleaving enzyme (CelOCE).^8^ These enzymes act synergistically to decompose the long chains of β-1,4-linked glucoses: while endoglucanases attack at random locations along the chain’s length to generate new ends, cellobiohydrolases attack these chain ends to generate cellobiose, which is, in turn, hydrolyzed by β-glucosidases. The latter process releases glucose as a product, which in turn can be fermented by microorganisms to generate bioethanol. As such, β-glucosidases play a key role in bioethanol technology. However, the main drawback of β-glucosidases is the fact that their product, glucose, is an inhibitor of enzymatic activity to most industrially important β-glucosidases.^9,10^ Consequently, the hydrolysis of cellobiose by β-glucosidases is a critical rate-limiting factor in the overall process of cellulose conversion.^7^ Remarkably, some β-glucosidases are tolerant and even stimulated by the presence of glucose.^11^ These enzymes have been shown to improve the hydrolysis of lignocellulosic biomass, but the mechanisms involved in such a distinct glucose dependence of β-glucosidases are not yet fully understood.^12^

Most glucose tolerant β-glucosidases belong to the glycoside hydrolase family 1 (GH1),^11^ with a deep substrate-binding cleft, usually narrower than in nontolerant enzymes, which has been suggested to hinder glucose accessibility to the active site.^13^ This may act as a first layer of glucose tolerance in many enzymes, but several other mechanisms have been suggested to explain this feature, including the hydrophobicity and flexibility of specific regions of the active cleft,^14,15^ the levels of transglycosylation activity,^16^ allosteric effects,^17–19^ and nonproductive binding of substrate.^20^ A recent review on the subject, highlighted that both glucose tolerance and stimulation are features that may stem from a variety of processes besides the ones already mentioned here,^21^ and, curiously, an efficient mechanism for glucose tolerance present in a specific enzyme may have no effect in a different one. For instance, the β-glucosidase from *Halothermothrix orenii* is stimulated by glucose, but this is completely independent from its transglycosylation activity,^15^ while in several similar enzymes the glucose tolerance and transglycosylation are intertwined.^16,22–24^

In this work, we focus on the β-glucosidase from filamentous fungi *Humicola insolens* (*Hi*Bgl), an enzyme from glycosyl hydrolase family GH1 that is not only tolerant to glucose, but also stimulated by it, which makes it remarkably suitable for industrial applications.^23^ We applied molecular dynamics (MD) simulations to assess atomistic details and thermodynamic features underlying product tolerance and stimulation in *Hi*Bgl. Our study builds upon recent works that focused on glucose dissociation mechanism and solvation structures in the *Hi*Bgl enzyme.^25,26^

## Methods

### Systems Setup and Simulation Details

In this study, we aimed to explore the behavior of *Hi*Bgl under different conditions. We have simulated different systems, either as independent multiple replicas or as single multi-μs runs in some cases, as summarized in Table 1.

**Table 1:** Details of the MD Simulations Performed in This Work

system	description	simulation time
A	*Hi*Bgl + cellobiose at subsites −1 and +1	3 × 500 ns
B	*Hi*Bgl + glucose at subsite −1	3 × 500 ns
C	*Hi*Bgl + glucose at subsite +2	5 × up to 100 ns
D	*Hi*Bgl without ligand	1 × 500 ns
E	*Hi*Bgl bound to two glucose molecules at subsites −1 and +1	5 × up to 500 ns
F	*Hi*Bgl + cellobiose in a 200 mM aqueous glucose solution	1 × 3.0 μs
G	*Hi*Bgl + glucose at subsite +2 in a 200 mM aqueous glucose solution	1 × 3.0 μs

Throughout this work, subsite −1 refers to the set of enzyme residues that interact with the first glucose unit toward the nonreducing end, adjacent to the glycosidic bond to be cleaved. Subsites +1 and +2 comprise the residues that interact with the first and second glucose units, respectively, on the reducing end side of the glycosidic bond targeted for cleavage. Subsite −1 comprises residues Gln17, His120, Trp121, Asn165, Glu166, Tyr308, Glu377, Trp427, Glu434, Trp435, and Phe443; subsite +1 comprises residues Cys169, Asn235, Tyr308, and Trp349; and subsite +2 comprises residues Trp168, Leu173, Tyr179, Phe348, and Trp349.

All systems were built starting from the crystal structure of *Hi*Bgl deposited on the Protein Data Bank (PDB) under the code 4MDP,^13^ after removal of the glycerol molecule located at subsite −1 and the glucose bound to subsite +2 (except in systems C and G). All crystallographic water molecules were retained, except the three molecules that appear at subsite −1. Water molecules numbered 601, 652, and 864 in PDB ID 4MDP were removed to make room for glucose and cellobiose accommodation. The cellobiose molecule of systems A and F was taken from the cellotetraose molecule available in the crystal structure of the rice BGlu1 (PDB ID 3F5J) after structural alignment with *Hi*Bgl.^27^ The glucose molecule of system B was taken from the crystal structure of the metagenomic Tf2d2 β-glucosidase (PDB ID 3WH6) after structural alignment with *Hi*Bgl.^22^ In systems C and G, the glucose molecule attached to subsite +2 was kept from the crystal structure of *Hi*Bgl (4MDP). The two glucose molecules of system E were obtained from the cellobiose molecule of system A after removing the glycosidic bond.

The protonation states of titratable residues were assigned with H++ considering pH 6.0.^28^ Except for the catalytic acid Glu166, which was considered protonated, all Glu and Asp residues were considered in their deprotonated states. His99, His184, and His315 residues were protonated at both N atoms; His120, His160, His211, and His353 residues were protonated only at the Nε atom; and His307 residue was protonated only at the Nδ atom.

Systems setup and MD simulations were carried out using Amber 16.^29^ The force fields used to describe the protein and the carbohydrates molecules were ff14SB^30^ and GLYCAM06,^31^ respectively, while the TIP3P water model^32^ was used to describe the solvent, with paddings of 13 Å in every direction of a rectangular box. Packmol^33^ was used to generate the 200 mM aqueous solution of glucose (100 glucose molecules + 19000 water molecules) for systems F and G, while all other systems were solvated with water using the LEaP module in Amber. Finally, 9 sodium ions were added in all simulation boxes to render the systems electrically neutral.

Periodic boundary conditions were employed and electrostatic interactions were evaluated with particle mesh Ewald.^34^ Short-range interactions were truncated at the distance cutoff of 8 Å. The temperature was maintained at 310 K in all simulations with the Langevin thermostat and the pressure was kept at 1.0 bar with the Berendsen barostat, as implemented in Amber 16.^29^ Bonds involving hydrogen atoms were constrained at their equilibrium lengths and a 2 fs time step was used to integrate the equations of motion. The initial geometries of all systems were energy minimized for 1000 steps using the steepest descent algorithm in the first 500 steps and conjugate gradients in the following 500 steps. An NPT equilibration step was carried out afterward for 200 ps with harmonic restraints of 50 kcal mol^–1^ Å^–2^ on all protein and carbohydrate atoms. Afterward, another cycle of minimization and equilibration was conducted, but this time with 1000 steps of steepest descent followed by 1500 steps of conjugate gradients, which were in turn followed by 200 ps of equilibration with the same harmonic restraints applied only on the α-carbons of *Hi*Bgl. At last, and before production, a third NPT equilibration was run without any restraints for 1.0 ns. For production runs, all systems ran in the NVT ensemble according to the simulation times listed in Table 1. Analyses were carried out with Cpptraj,^35^ VMD,^36^ and in-house codes.

### Potential of Mean Force Calculations

Umbrella sampling (US)^37^ simulations were employed to obtain the potential of mean force (PMF) associated with the unbinding of both cellobiose and glucose from *Hi*Bgl. Such simulations will henceforth be referred to as systems A_US_ and B_US_ for brevity. The PMFs were computed along the reaction coordinate defined as the distance between the CD atom of the catalytic residue Glu377 and the C1 atom of the glucosyl residue bound to the subsite +1 and – 1, for systems A and B, respectively. Biasing harmonic potentials of the form *V* = *k*(ξ – ξ_0_)^2^, where ξ is the reaction coordinate, were employed to sample the configuration spaces. For system A_US_, we split the reaction coordinate domain into 62 windows, each centered at values between 3.5 Å and 30.0 Å, with increments of 0.3 Å up until the 21st window, when the increments became 0.5 Å. The force constant was set to *k* = 20 kcal mol^–1^ Å^–2^. For system B_US_, we considered 20 windows between 3.5 Å and 9.2 Å with increments of 0.3 Å and force constant of *k* = 20 kcal mol^–1^ Å^–2^, followed by 22 windows between 9.5 Å and 20.0 Å with increments of 0.5 Å and force constant of *k* = 5 kcal mol^–1^ Å^–2^. Two additional windows centered at 5.4 Å (*k* = 50 kcal mol^–1^ Å^–2^) and 5.5 Å (*k* = 150 kcal mol^–1^ Å^–2^) were also included. These schemes proved sufficient to generate overlapping biased histograms ().

Restrained MD simulations were carried out within each window after 500 steps of energy minimization (steepest descent and conjugate gradient split equally) and 100 ps of equilibration (not considered in the PMF calculation). For systems A_US_ and B_US_, 300 and 500 ns for each window, respectively, were necessary to converge the PMFs (Figure S1). The initial coordinates for the first 20 windows in systems A_US_ and B_US_ were taken from the third unbiased equilibration step mentioned above for systems A and B, respectively, whereas the initial coordinates for the remaining windows were taken from the last saved configuration of the 20th window in each system. Finally, the Weighted Histogram Analysis Method (WHAM) was employed to obtain the PMFs from the biased histograms.^38,39^ Error bars were obtained through bootstrap error analysis.

## Results and Discussion

### *Hi*Bgl Bound to Cellobiose and Glucose

We first sought to compare the dynamics of *Hi*Bgl when in the presence of the cellobiose substrate (system A) and in the presence of glucose (system B). In both systems, we observed no spontaneous dissociation of either ligand. The distance between the CD atom of the catalytic nucleophile Glu377 and the C1 atom of the glucose bound to subsite –1 was computed to assess the relative position of the ligand in the active site (Figure S2). While such distance is 4.3 Å in the initial structure, it fluctuates between 4 and 6.5 Å in system A (cellobiose-bound), and between 3 and 5 Å in system B (glucose-bound). Thus, only local fluctuations of the ligands were observed in our simulations, with no dissociation. In addition, no large-scale structural fluctuations were observed for *Hi*Bgl, as revealed by the RMSD’s (root mean squared deviation) of the α-carbons relative to the crystal structure, which remain mostly below 1.2 Å throughout the simulations (Figure S3).

When glucose acts as a competitive inhibitor at subsite −1 (system B), we observe that *Hi*Bgl’s substrate binding site may experience mild, but relevant structural changes. Conversely, such events do not occur with *Hi*Bgl bound cellobiose (system A) or in the absence of ligands (system D).

Figure 1A shows a snapshot of the active site of *Hi*Bgl bound to cellobiose (system A), where it can be seen that Trp349 is part of subsite +1. Figure 1B shows the distance between the C1 atom of the glucosyl residue located at subsite −1 and the CH2 atom of Trp349 (represented by a dotted line connecting these atoms in Figure 1A). This distance fluctuates around 5 Å in all of the three MD simulations performed for this system and indicates that Trp349 remains in place when cellobiose is bound. Figure 1C shows a snapshot of *Hi*Bgl bound to glucose (system B) in which Trp349 (subsite +1) assumes a conformation similar to what is seen in the crystal structure. In Figure 1D, another snapshot is shown in which the side chain of Trp349 flips toward the solvent exposed area, interacting with Phe348, thus disassembling subsite +1. The time evolution of the distance between the C1 atom of glucose and the CH_2_ atom of Trp349, shown in Figure 1E for system B, indicates that Trp349 changes conformation toward Phe348 in all three simulations, where such distance reaches values of up to 20 Å. This opens room for water penetration and solvation of glucose (Figure S4), which could contribute to a low affinity of glucose for subsite −1. The fact that the subsite +1 remains intact when cellobiose is bound is consistent with its high affinity for the enzyme. When the binding site is vacant (system D), the subsite +1 remains intact in a single 500 ns-long simulation (Figure S5), which means that the enzyme is in the proper conformation for substrate binding. Also, this result shows that Trp349 conformational change is an effect dependent on the presence of glucose at subsite –1. We therefore suggest that Trp349 side chain flipping observed at subsite +1 for *Hi*Bgl bound to glucose at subsite –1 might be involved in promoting glucose expulsion from the binding site, sustaining this enzyme’s resistance to product inhibition.

**Figure 1:**
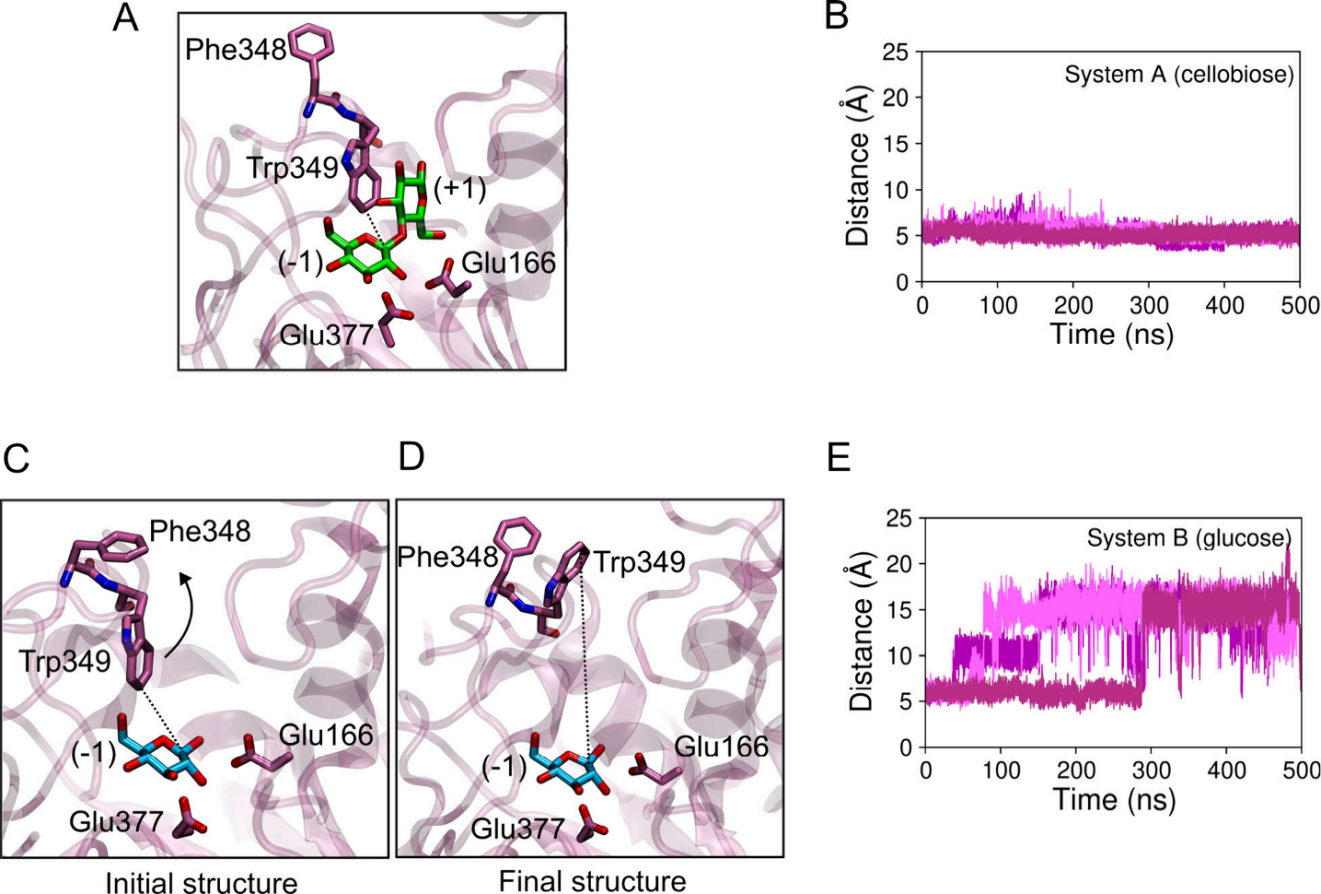
(A) Snapshot of *Hi*Bgl + cellobiose (system A) showing residue Trp349 as part of subsite +1. (B) Distance between the C1 atom of the glucosyl residue of cellobiose (in green) located at subsite −1 and the CH_2_ atom of Trp349, represented as a dotted line in panel A. Snapshot of *Hi*Bgl + glucose (system B) with subsite +1 (C) intact and (D) disassembled. (E) Distance between the C1 atom of the glucose (in cyan) and the CH2 atom of Trp349, represented as a dotted line in panels C and D. In panels B and E, different colors represent different independent MD simulations.

Next, we simulated *Hi*Bgl with glucose bound to subsite +2 (system C), which is the location where it binds the enzyme in the crystal structure.^13^ The goal of this simulation was to assess how stable the binding of glucose to subsite +2 is and whether this subsite could prevent glucose from reaching the cellobiose binding site (subsites −1 and +1), which would result in competitive inhibition. In all 5 independent simulations, we found that glucose initially at subsite +2 readily moves to subsite +1 and establishes CH−π interactions with Trp349 during the equilibration phases, indicating that, in solution, affinity of glucose for subsite +2 is likely low. The CH−π interactions with Trp349 are assisted by hydrogen bonds between glucose and residue Asp237, which has been suggested to play roles in glucose dissociation.^25^ Figure 2 shows the minimum distance between glucose and residue Trp349 versus time in each of these simulations. We observe that, although Trp349 is able to hold the glucose molecule for a while, binding is not effective, and the glucose leaves to the bulk of solution after tens of nanoseconds, reinforcing the view that the binding pocket of *Hi*Bgl is likely unable to stably harbor glucose in the subsite +2, thus unable to prevent it from reaching the cellobiose binding site. Rather, glucose is more likely to go into the solution once it is out of subsite −1.

**Figure 2:**
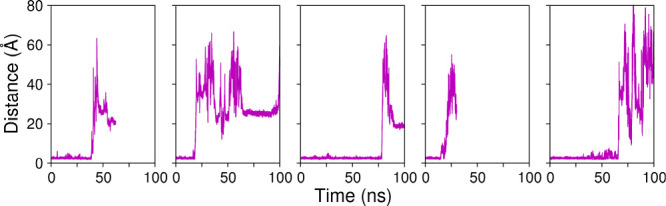
Distance between glucose and Trp349 in 5 independent simulations of system C, where *Hi*Bgl is complexed to glucose initially at the subsite +2. The short initial distances correspond to the glucose molecule bound to subsite +1, where Trp349 is located. We notice that in less than 100 ns the glucose molecule exits the binding pocket, as indicated by the abrupt increase in the distance.

### Potential of Mean Forces of Cellobiose and Glucose Dissociation

Since no dissociation of glucose from subsite −1 was observed in our simulations, we employed Umbrella Sampling to drive this event and compute the unbinding PMF of both cellobiose and glucose. For cellobiose unbinding, we employed a reaction coordinate defined as the distance between the CD atom of the catalytic nucleophile Glu377 and the C1 atom of the glucosyl residue initially bound to subsite +1 (Figure 3A). Figure 3B shows the PMF for cellobiose dissociation, from which we obtain a binding free energy of −12.5 kcal/mol (as shown in Figure 3B, error bars are below the thermal energy of ∼0.6 kcal/mol). For glucose unbinding, the reaction coordinate was defined as the distance between the CD atom of the catalytic nucleophile Glu377 and the C1 atom of the glucose, initially bound to subsite –1 (Figure 3C). As shown in Figure 3D, the computed PMF for glucose unbinding yields a binding free energy for glucose of −4.3 kcal/mol (error bars are below thermal energy). This indicates that *Hi*Bgl has higher affinity for its cellobiose substrate than for its glucose product. This result therefore suggests that, when both cellobiose and glucose are present in the medium, cellobiose will preferentially bind *Hi*Bgl and glucose would not be a competitive inhibitor. This difference in substrate/product binding helps explain why *Hi*Bgl is tolerant to the glucose product.

**Figure 3:**
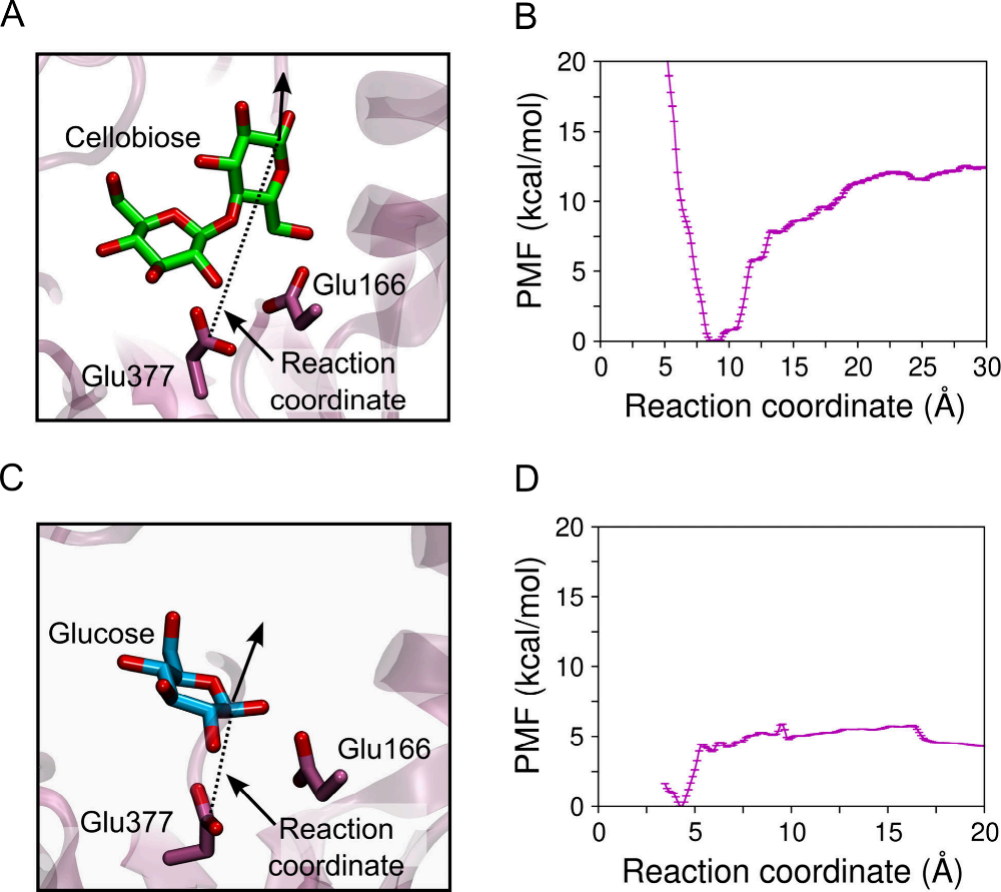
Scheme showing the reaction coordinates employed to compute the PMF of (A) cellobiose and (C) glucose dissociation from *Hi*Bgl. PMF of (B) cellobiose and (D) glucose dissociation. The enzyme’s affinity for its substrate is higher than for its product, which is consistent with the fact that *Hi*Bgl is tolerant to, and not inhibited by, glucose. Error bars were computed by bootstrap error analysis, and are much smaller than the thermal energy.

Interestingly, we observe that while the PMF of cellulose dissociation increases continuously from the bound to the unbound state, the PMF of glucose dissociation exhibits an initial abrupt increase, where glucose exits subsite −1, and then it remains nearly flat as the reaction coordinate increases. Thus, when cellobiose approaches the entrance of the binding pocket, there is a driving force that propels it toward the active site. In contrast, there is no such a driving force as glucose travels along the binding pocket, except when it gets sufficiently close to subsite −1 for binding. This is consistent with our MD simulations starting from glucose at subsite +2 (Figure 2), where glucose dissociation is consistently observed, with only weak interactions with the binding-pocket residues.

### *Hi*Bgl Immersed in an Aqueous Glucose Solution

Systems F and G, where *Hi*Bgl is bound to cellobiose at subsites −1/+1 and glucose at subsite +2, respectively, are immersed in a 200 mM aqueous glucose solution, which resembles a typical condition of glucose stimulation for *Hi*Bgl.^40^ Figure 4A depicts the simulation box containing *Hi*Bgl in the aqueous glucose solution. Such high concentration of glucose is common in reaction tanks for second generation ethanol production, where the hydrolysis products of polysaccharides from lignocellulosic biomass accumulate fast.^23^

**Figure 4:**
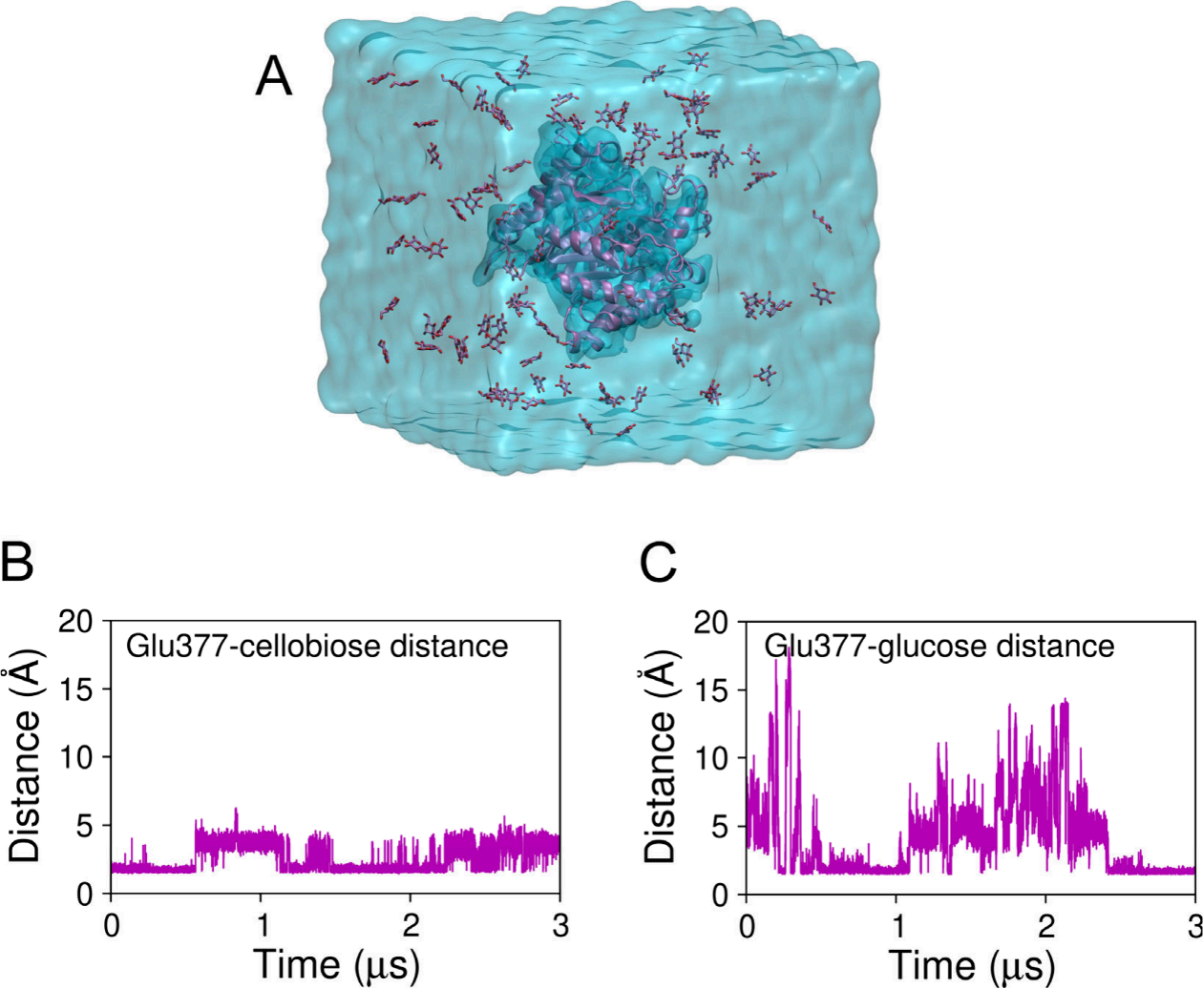
(A) Simulation box showing the aqueous solution of glucose surrounding *Hi*Bgl in systems F and G. Distance between the CD atom of Glu377 and (B) cellobiose in system F and (C) the closest glucose molecule in system G. While cellobiose remains bound to its binding site in *Hi*Bgl, glucose binds only temporarily, which is consistent with the product tolerance of the enzyme.

*Hi*Bgl in systems F and G went through 3.0 μs of MD simulation. The RMSDs for the α-carbons relative to the crystal structure reveal that the enzyme in these systems remains stable, with RMSD values below 1.5 Å (Figure S6), which is slightly higher than the displacements in systems A and B (mostly below 1.2 Å, Figure S3). This feature of glucose solution has been reported for another β-glucosidase.^41^ Figure 4B shows the distance between the CD atom of the catalytic residue Glu377 and the closest atom of cellobiose, revealing that the substrate remains bound to the enzyme throughout the MD simulation in glucose solution. Figure 4C shows the distance of the CD atom of Glu377 and the closest glucose residue. We observe that glucose can reach the subsite −1 from the solution when the Glu377–glucose distance fluctuates around the lowest value of ∼1.7 Å. However, glucose binding is not stable, as it is observed to be released back to the solution during the simulations. These results indicate that, when immersed in a glucose solution, *Hi*Bgl still retains high affinity for cellobiose and a much lower affinity for glucose, consistent with the PMF calculations described above for *Hi*Bgl in aqueous solutions.

### Direct Glucose–Cellobiose Interactions

*Hi*Bgl not only is tolerant to, but can be also stimulated by the reaction product.^23^ The role of glucose (i.e., inhibition, tolerance, and/or stimulation) has been suggested to be dependent on the location of its binding site relative to the substrate (cellobiose) binding site.^42^ One hypothesis is that β-glucosidases stimulated by product exhibit additional binding sites where glucose binds with high affinity and interacts favorably with the substrate, thereby either increasing its affinity for the enzyme or helping stabilize transition states. On the other hand, it has been proposed that β-glucosidases that are inhibited by the reaction product would not exhibit such additional binding sites and glucose would compete with the substrate for the active site.^42^

Analysis of the MD simulation of system F showed that glucose can penetrate the binding pocket and interact directly with cellobiose. Figure 5A shows one such configuration, where a glucose molecule is pictured hydrogen bonding the cellobiose substrate. This interaction may have an effect on the energetics of the hydrolysis reaction or prevent dissociation of the glucose product after the glycosylation step of the reaction mechanism, favoring transglycosylation reactions. Figure 5B shows that the distance between cellobiose and the closest glucose molecule fluctuates significantly, indicating that glucose molecules can switch from *Hi*Bgl-bound to solution states in a time scale of hundreds of nanoseconds. After ∼1.2 μs of MD simulation, we observe a stabilization of this distance, suggesting that a glucose molecule might have better accommodated around the cellobiose, with less exchange with the bulk molecules. Considering all the simulation time, Figure 5C shows the density of probability of the cellobiose–glucose distance. The sharp peak centered around ∼1.9 Å indicates that there is a glucose molecule hydrogen bonding the cellobiose most of the time, potentially stimulating *Hi*Bgl activity.

**Figure 5:**
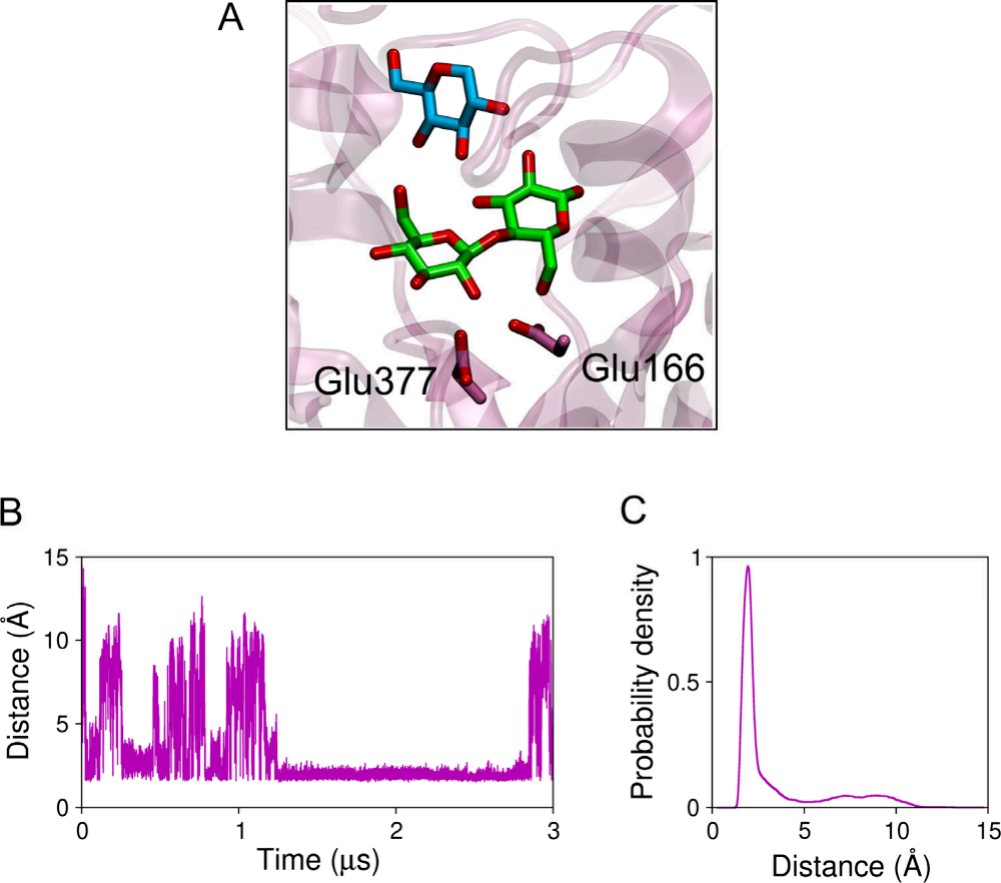
Direct interaction of glucose and cellobiose. (A) Snapshot showing a glucose molecule from the solution (in cyan) hydrogen bonding the cellobiose (in green) in the active site of *Hi*Bgl. The catalytic residues are shown. (B) Distance between cellobiose and the closest glucose molecule along the simulation. (C) Probability density of the cellobiose–glucose distance.

### Glucose Accessibility to the Binding Pocket

We further observed that the glucose accessibility to the *Hi*Bgl binding pocket increases when the enzyme is immersed in the glucose-rich solution, compared to when there are no glucose molecules around. Figure 6A shows two superposed snapshots of *Hi*Bgl highlighting the loops containing residues Tyr179 and Phe348 (represented by spheres centered at their α-carbons). These loops are located at the entrance of the *Hi*Bgl binding pocket, and the distance between the α-carbons of Tyr179 and Phe348 is 13.9 Å in the crystal structure. The MD simulations revealed that the left loop, where Phe348 is located, spans open and closed conformations that can modulate the accessibility of the active site. Figure 6B shows the distribution of the interloop distance, measured as the distance between the α-carbon of residues Tyr179 and Phe348, for *Hi*Bgl + cellobiose in aqueous solution (system A) and immersed in glucose solution (system F). We observe that while the Tyr179–Phe348 distance fluctuates around 14.6 Å in the presence of water (system A), it fluctuates around a larger range of values in the presence of the glucose-rich solution, with a bimodal distribution featuring a primary peak at 14.0 Å (slightly more compact than in water) and a secondary peak at 16.5 Å (wider than in water). This shows that *Hi*Bgl responds to the glucose-rich solution with a more flexible binding site entrance, which may facilitate the access of glucose to the cellobiose located at the bottom of the pocket.

**Figure 6:**
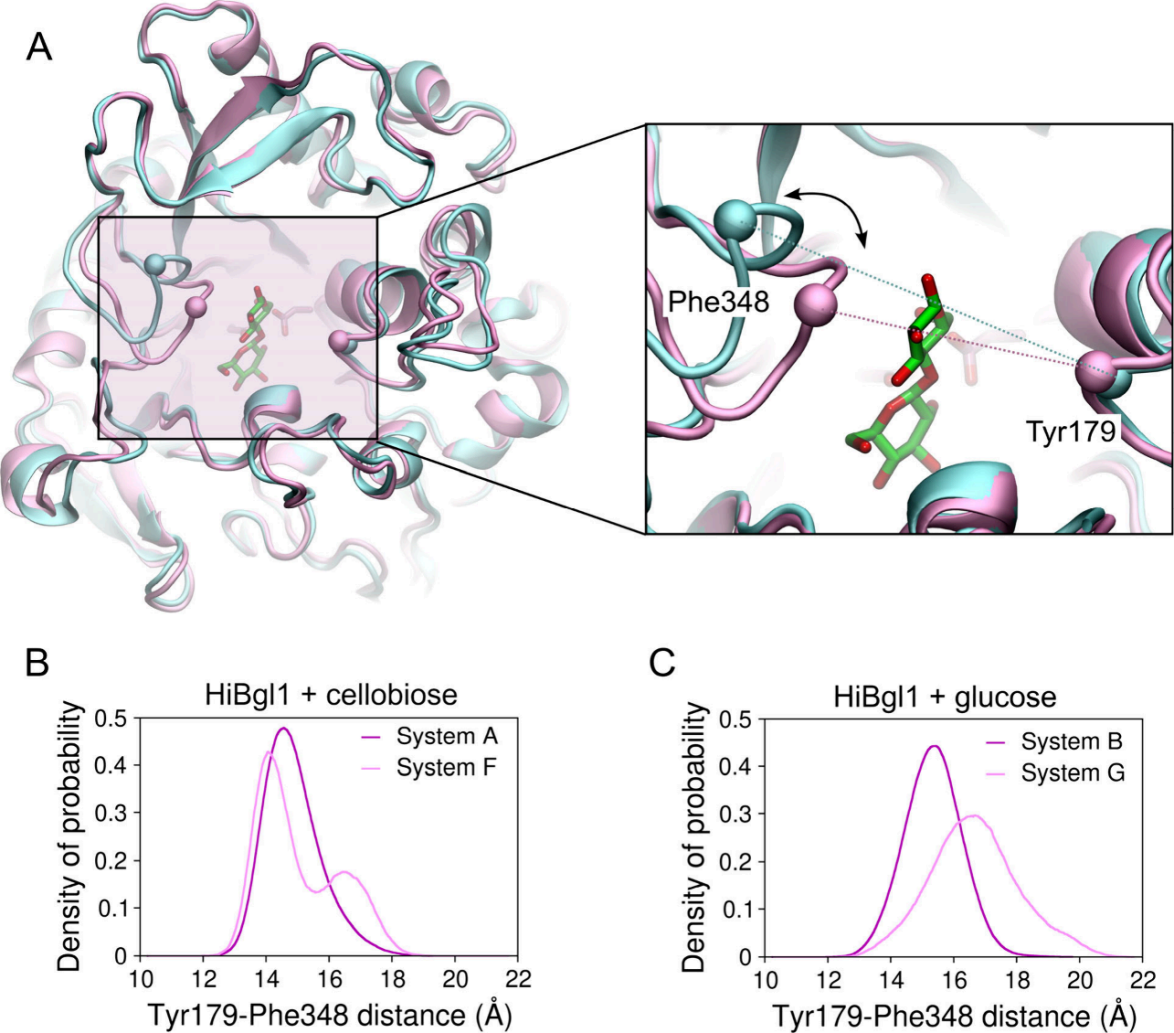
Open and closed conformations at the *Hi*Bgl binding site entrance. (A) Snapshots of *Hi*Bgl in system A showing the closed (pink) and open (cyan) states. The spheres represent the α-carbon of residues Tyr179 and Phe348. The dotted lines on the right panel indicate the distance employed to characterize open/closed conformations. Distribution of the distance between the α-carbons of Tyr179 and Phe348 for systems (B) A and F and (C) B and G.

When it comes to *Hi*Bgl bound to glucose (system B), as shown in Figure 6C, we first notice that it exhibits slightly wider binding site entrance (Tyr179–Phe348 distance peaked at ∼15.4 Å) than the corresponding system A with cellobiose bound (Tyr179–Phe348 distance peaked at ∼14.6 Å). This suggests that *Hi*Bgl bound to glucose at subsite −1 exhibits motions that could help product expulsion from the binding pocket. When *Hi*Bgl is immersed in a glucose-rich solution without cellobiose (system G), we observe that the Tyr179–Phe348 distances reach values substantially larger (up to ∼20 Å with peak at ∼16.7 Å) than those exhibited by system B, indicating a wider amplitude of fluctuations induced by the glucose molecules in solution, which can close and open the binding pocket.

Fluctuations of the binding pocket volume were also observed in the study of *Hi*Bgl in glucose aqueous solution in absence of cellobiose, but more compact structures were obtained in a shorter time scale (1.0 μs).^26^ In another study, based on homology-modeled structures, extensive accelerated MD simulations suggested that mutations which increase glucose tolerance in a nontolerant β-glucosidase, increase the plasticity of the substrate-binding pocket.^43^ Broadening of the binding pocket entrance was also observed at high glucose concentrations in the *Halothermothrix orenii* family GH1 β-glucosidase.^15,44,45^ Taken together, these results indicate that the presence of glucose, either in solution or at subsite −1, has the effect of widening the binding site entrance and, therefore, increasing glucose exchange with the bulk. This could (i) help glucose entrance to potentially stimulate the catalytic activity on cellobiose and (ii) help glucose expelling in the absence of cellobiose, rendering the enzyme tolerant to its product. Moreover, by identifying patterns of behavior of *Hi*Bgl in other enzymes, it might be possible to envisage mutations that improve even further the activity of this biocatalyst and the yields of environmentally friendly fuels.

### Secondary Glucose Binding Sites

Several studies have pointed to the potential presence of allosteric effects influencing the glucose-stimulated activity of GH1 β-glucosidases.^17,18,23,40,46^ To detect potential allosteric sites, we searched for amino acid residues in the enzyme structure that were frequently interacting with glucose molecules from the solution. Figure 7A,B shows the frequency of having at least one glucose molecule within 3 Å of a given residue, for systems F and G, respectively.

**Figure 7:**
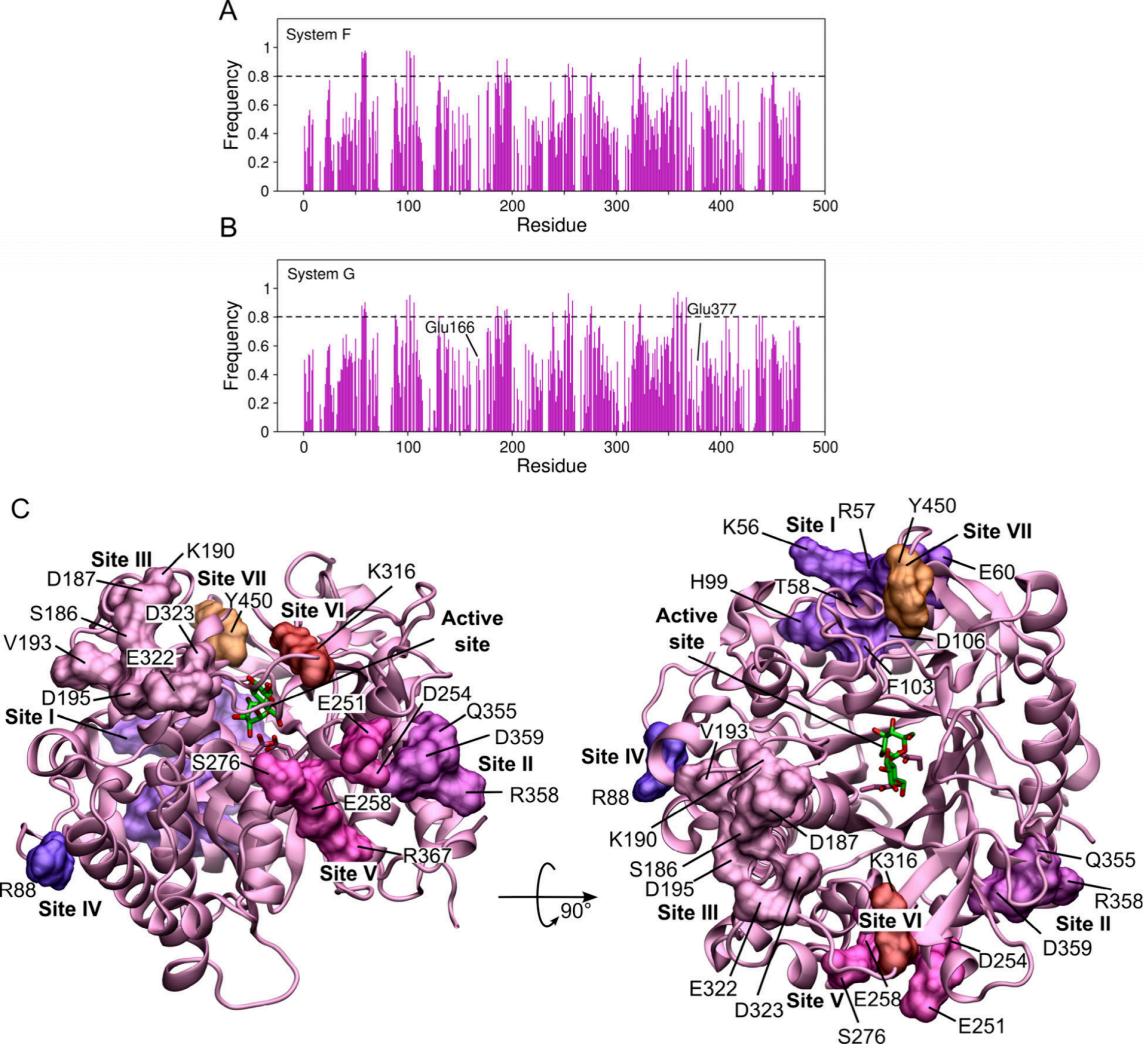
Secondary glucose binding sites. Frequency that there is a glucose molecule within 3 Å of each residue of *Hi*Bgl in (A) system F and in (B) system G. Residues that interacted with glucose more than 80% of the time (dashed lines) were clustered in different secondary binding sites. (C) Spatial distribution of the secondary binding sites on the *Hi*Bgl’s surface.

Interactions of glucose with the residues of *Hi*Bgl exhibited approximately the same frequency in systems F and G, indicating that the presence of cellobiose does not have an effect on secondary binding of glucose (Figures 7A and 7B). We determined secondary glucose binding sites on the enzyme’s surface by selecting those residues for which interaction with glucose was present more than 80% of the time and by clustering the spatially close ones. From this approach, we found 7 secondary glucose binding sites on *Hi*Bgl’s surface, henceforth numbered I–VII (Figure 7C and Table 2). Most of the residues are either charged or polar, so the interactions with glucose are mainly through hydrogen bonding. This finding has been recently reported in another computational study on *Hi*Bgl through the analysis of minimum-distance distribution functions.^26^

**Table 2:** Secondary Glucose Binding Sites Found by Simulation of System F

binding site	residues	distance to the active site^a^ (Å)
I	Lys56, Arg57, Thr58, Lys59, Glu60, His99, Lys102, Phe103, and Asp106	21.9 ± 0.7
II	Gln355, Arg358, and Asp359	20.1 ± 0.6
III	Ser186, Asp187, Lys190, Val193, Asp195, Glu322, and Asp323	26.5 ± 0.4
IV	Arg88	28.9 ± 0.6
V	Glu251, Asp254, Glu258, Ser276, and Arg367	23.2 ± 0.5
VI	Lys316	29.0 ± 0.8
VII	Tyr450	20.2 ± 0.5

a The minimum distance between the CD atom of Glu377 and the center of mass of the secondary binding sites.

Previous studies have suggested that allostery in *Hi*Bgl probably stems from regions close to the substrate binding site.^11^ Our results indicate that sites II and VII are the closest ones from the active site (Table 2) and, therefore, we suggest them to be considered in future site-mutagenesis studies. This analysis allowed us to propose potential allosteric sites that would be triggered by glucose in *Hi*Bgl’s surface, potentially stimulating its activity. A previous computational study evaluated the presence of secondary glucose binding sites for uncompetitive inhibition of β-glucosidase H0HC94 *Agrobacterium tumefaciens* 5A. Six secondary binding sites were found using MD simulations of this enzyme immersed in a 0.8 M glucose solution.^41^ Additional glucose binding sites were found in mutants with higher tolerance to glucose.^47^ Likewise, a combination of docking and MD simulations revealed monosaccharide secondary binding sites on the surface of the *Termotoga petrophila* β-glucosidase 1, which was suggested to play roles in increasing substrate accessibility to the enzyme’s active site.^48^

Ramos and Martínez performed a detailed analysis of binding of glucose to *Hi*Bgl residues of the binding pocket based on minimum-distance distribution functions.^26^ In our analysis, we made no distinction based on the location of the residues, so we were able to capture those interactions that are established on the surface of the enzyme, far from the active site. Although we can observe that the glucose molecules interact with binding-pocket residues, the frequencies of these interactions are below 0.8, indicating that the secondary binding sites are able to hold glucose molecules more efficiently than the binding-pocket residues. In accordance with the study of Ramos and Martínez,^26^ our simulations also show that catalytic residues Glu166 and Glu377 are not among the residues with which glucose interacts more frequently in absence of cellobiose (Figure 7B), further corroborating the fact that *Hi*Bgl is tolerant to its product.

### Glucose Reorientation at Subsite +1: Insights into Transglycosylation

In order to study the behavior of the glucose product immediately after it is formed, we built and simulated system E, which consisted of *Hi*Bgl bound to a hydrolyzed cellobiose molecule, that is, a glucose molecule at subsite −1 and another at subsite +1. While glucose at subsite −1 remained tightly bound to *Hi*Bgl through interactions with residues Gln17, His120, Asn165, Glu166, Glu377, and Glu434, the other glucose molecule left subsite +1 to the bulk after ∼100, ∼30, and ∼200 ns in three independent simulations, and remained in place during two other 500 ns-long simulations, with no dissociation events detected. Overall, glucose at subsite +1 in system E exhibited higher residence times than in system C, where the single glucose molecule systematically left *Hi*Bgl in less than 100 ns (Figure 2). This suggests that glucose–glucose interactions played a role in retaining the glucose initially at subsite +1 for longer times. We analyzed such interactions to get structural insights into transglycosylation reactions that *Hi*Bgl is able to perform.

Using one of the MD simulations in which glucose did not dissociate within the 500 ns run, we measured distances between the C1 atom of glucose at subsite −1 and the atoms O2, O3, O4, and O6 of glucose at subsite +1 (Figure 8A,B). These distances are related to the formation of β-1,2, β-1,3, β-1,4, and β-1,6 bonds in transglycosylation reactions. At the very beginning of the simulation, the shortest distance among the four is that between C1 and O4, which is due to the fact that the starting configuration is that of a hydrolyzed cellobiose molecule. Nonetheless, we observe that glucose can rotate at subsite +1 and interact with the glucose at subsite −1, also through the O2, O3, and O6 atoms. Thus, the pair of atoms that exhibit the shortest distance changes over time (Figure 8A), suggesting that nucleophilic attack on carbon C1 at subsite −1 could happen by any of the four O atoms at subsite +1, leading to different transglycosylation products. Figure 8B shows the density of probability of each of the four distances, which reveals that all four distances exhibit a peak at 4 Å, even though the probabilities are higher for C1–O6 and C1–O4 interactions. Figure 8C–F show snapshots of the *Hi*Bgl active site with glucose at subsite −1 establishing different interactions with glucose at subsite +1. This analysis was performed for the other four independent MD simulations of system E and is shown in Figure S7, from which similar conclusions are drawn, although with poorer sampling in some cases. Altogether, these results tell us that, while −1 glucose is firmly bound in the active site, the +1 glucose molecule has freedom to rotate, which allows for the formation of multiple transglycosylation products (Figure 8C–F).

**Figure 8:**
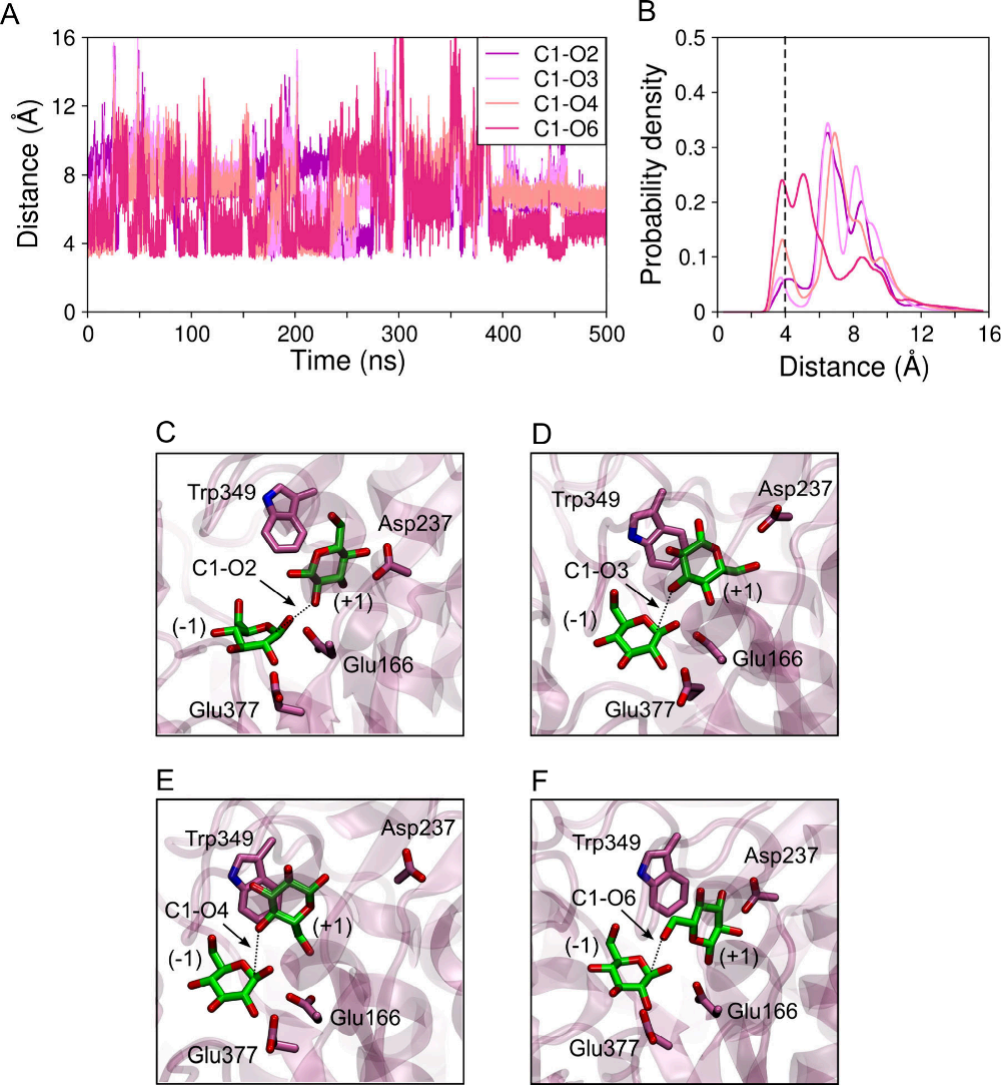
*Hi*Bgl bound to two glucose molecules at subsites −1 and +1 (system E). (A) Distance between the C1 atom of glucose bound to subsite −1 and the O2, O3, O4, and O6 atoms of the glucose bound to subsite +1 along a 500 ns long trajectory in which no dissociation was observed. (B) Probability density of the distances shown in panel (A). The dashed vertical line indicates minimum equilibrium distance between the pairs of atoms and their probabilities. (C–F) Representative snapshots showing different orientations of the glucose bound to subsite +1. The dotted lines connect atoms for which the distance is minimum in each case. Asp237 hydrogen bonds the glucose molecule in the configurations (C), (D), and (F).

Furthermore, we observed that residue Asp237 hydrogen bonds the glucose molecule in distinct orientations, allowing for formation of C1–O2, C1–O3, or C1–O6 bonds. This suggests that Asp237 is important for the transglycosylation activity of *Hi*Bgl by helping stabilize glucose orientations that lead to transglycosylation products. These results are consistent with the experimental findings showing that the mutation Asp237Val resulted in a lower transglycosylation/hydrolysis ratio in *Hi*Bgl, as well as lower stimulation by glucose and xylose compared to the wild type enzyme.^23^

It should be emphasized here that transglycosylation reactions occur from the glycosyl–enzyme intermediate, after nucleophilic attack of Glu377 on the anomeric carbon and cleavage of the glycosidic bond. In our simulations, we use a hydrolyzed cellobiose molecule to get insights into the possible orientations of glucose at subsite +1 relative to glucose at subsite −1. Therefore, the probability densities shown in Figure 8B should not be interpreted as probabilities of nucleophilic attacks for different transglycosylation products, nor as the expected ratios between different products. Rather, they should be taken only as an indicator that glucose can adopt different orientations at subsite −1, which enable transglycosylation reactions, which are often associated with glucose stimulation of the catalytic activities.^11^

## Conclusions

Our study explores *Hi*Bgl in different conditions and provides insights into the glucose tolerance and stimulation mechanisms in β-glucosidases. We show that the presence of glucose at subsite −1 shifts Trp349 and locally disassembles subsite +1, to potentially expel glucose and maintain its tolerance to product. Potential of mean force calculations reveal that the binding affinity of the cellobiose substrate is substantially higher than that of the glucose product, providing a thermodynamic basis for product tolerance. Using simulations of *Hi*Bgl immersed in aqueous glucose solutions, we further suggest that (i) glucose from the solution can interact directly with cellobiose, potentially having effects on the catalysis; (ii) the binding pocket becomes broader in the presence of glucose, allowing exchange with molecules from the bulk; (iii) glucose can strongly interact with 7 secondary binding sites on the enzyme surface, potentially acting as allosteric effector. Additionally, we observed that glucose at subsite +1 is able to reorient itself relatively to another glucose at subsite −1, which is a requisite to form multiple transglycosylation products. These insights provide a deeper understanding of *Hi*Bgl’s functional versatility and pave the way for targeted mutational studies to enhance its industrial applications, particularly in biofuel production and oligosaccharide synthesis.

## Supplementary Material





## Data Availability

Coordinates, topology, and input files used to generate the simulations are provided as part of the Supporting Information.

## Notes

Amber coordinates (INPCRD), topology (PRMTOP), and input files (IN) needed to reproduce the MD simulations of systems A–G, and the Umbrella Sampling calculations (ZIP)
Additional figures (PDF)
