# Supplementary material for: Computational Insights into Glucose Tolerance and Stimulation in a Family 1 β‑glucosidase
Source: J Chem Inf Model. 2025 Jul 1;65(13):7102–12. doi: 10.1021/acs.jcim.5c00922 (PMC12264956; doi:10.1021/acs.jcim.5c00922)
Supplement: Supplementary file 2 [file ci5c00922_si_002.pdf]

SUPPORTING INFORMATION

Computational Insights into Glucose Tolerance and Stimulation in a Family 1 β-glucosidase

Artur H. S. Dias[‡,§], Munir S. Skaf[‡,§], and Rodrigo L. Silveira[†,*]

† Institute of Chemistry, Federal University of Rio de Janeiro, Av. Athos da Silveira Ramos 149, 21941-909, Rio de Janeiro, RJ, Brazil

‡ Institute of Chemistry, University of Campinas, Rua Monteiro Lobato 270, 13083-862, Campinas, Sao Paulo, Brazil

§ Center for Computing in Engineering and Sciences, University of Campinas, Rua Monteiro Lobato 270, 13083-862, Campinas, Sao Paulo, Brazil

*Corresponding author. E-mail: rodrigo.silveira@iq.ufrj.br

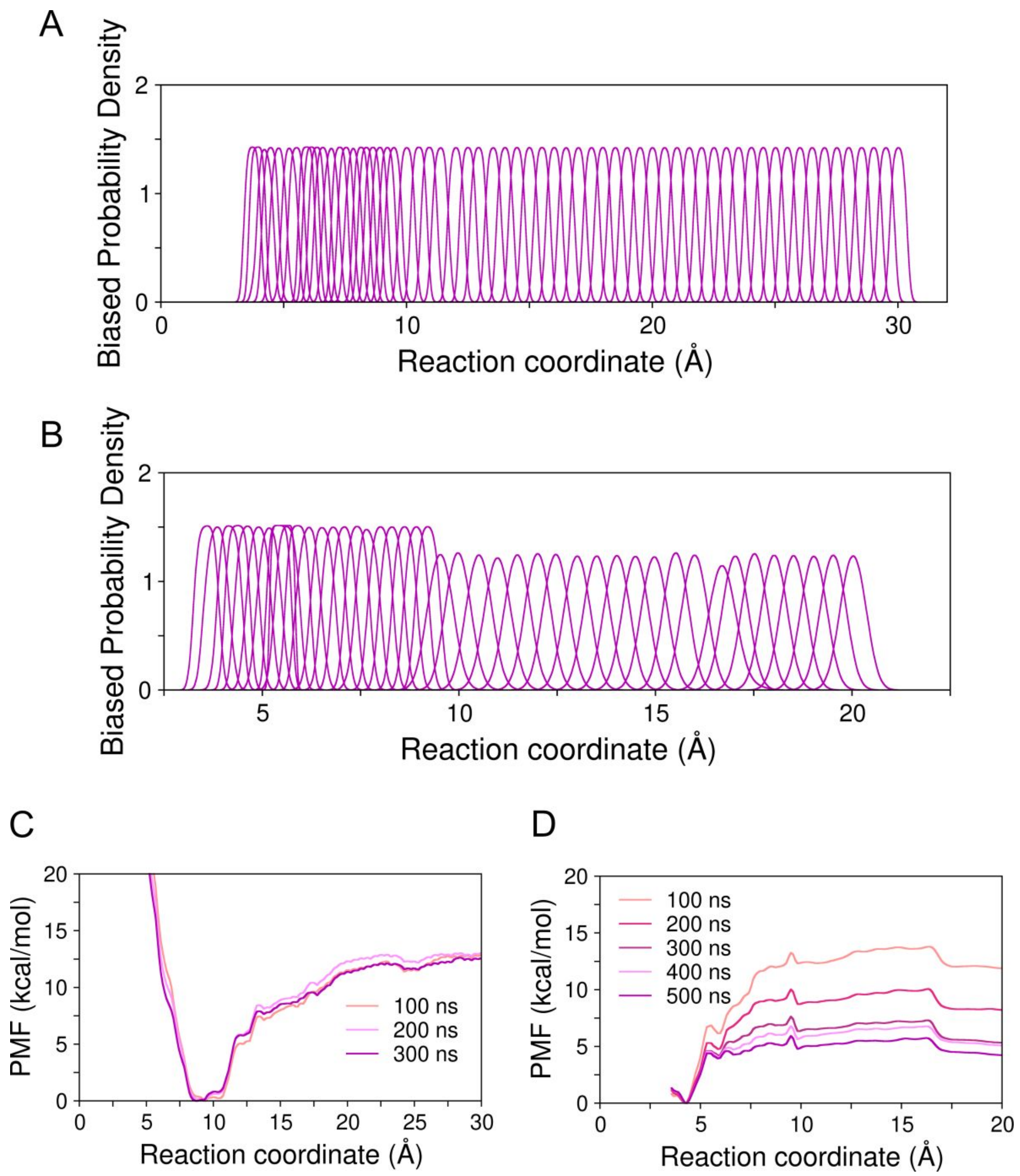

Figure S1. Biased histograms for systems (A) A_{US} and (B) B_{US} showing overlap between adjacent windows. (C) PMF of cellobiose dissociation in system A_{US} computed in different simulation times, showing convergence in 300 ns of MD simulation per window. (D) PMF of glucose dissociation in system B_{US} computed in different simulation times, showing that convergence has been achieved with 500 ns of MD simulation per window.

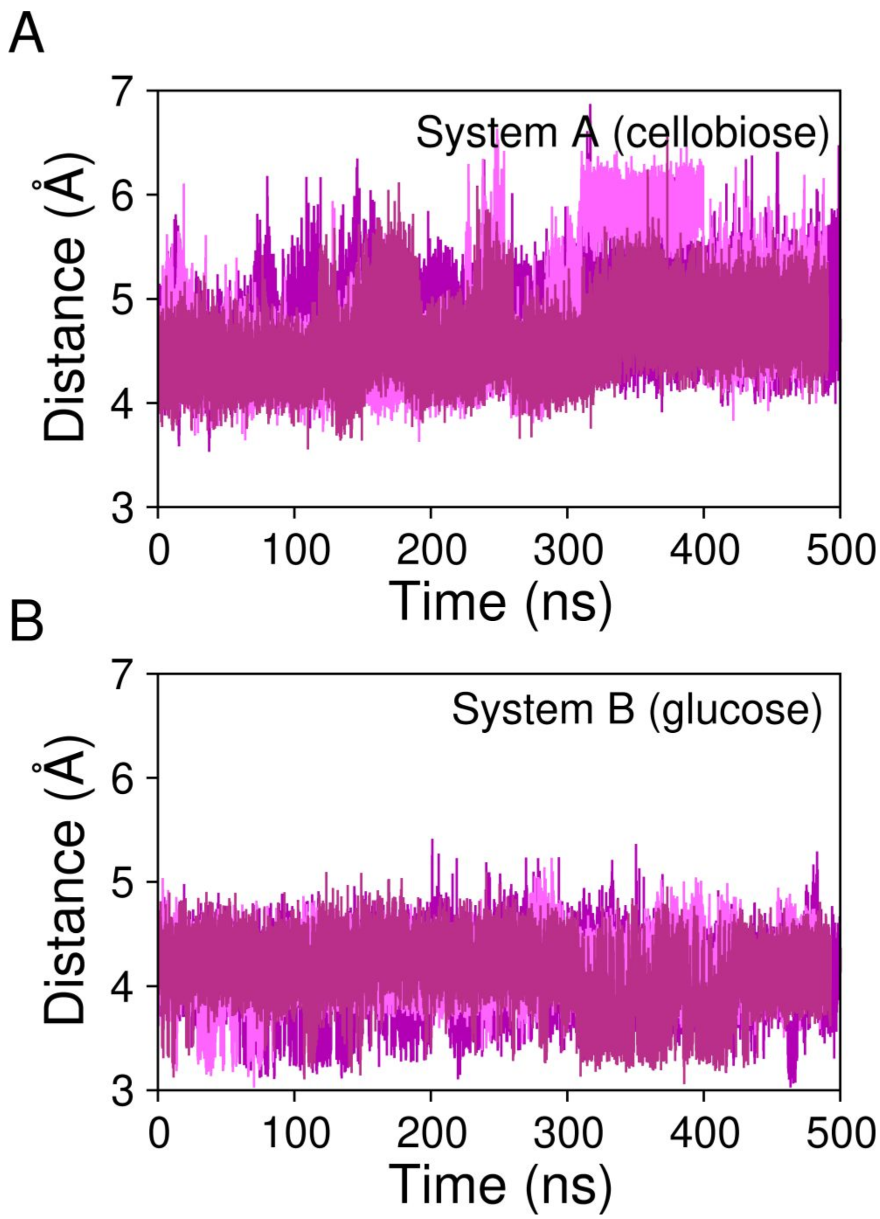

Figure S2. Distance between the CD atom of the catalytic base Glu377 and the C1 atom of the (A) glucosyl unit of cellobiose bound to subsite -1 (system A) and (B) glucose bound to subsite –1 (system B). The time evolution of these distances shows that neither cellobiose nor glucose left the active site of *Hi*Bgl during our simulations. Different colors mean different independent simulations.

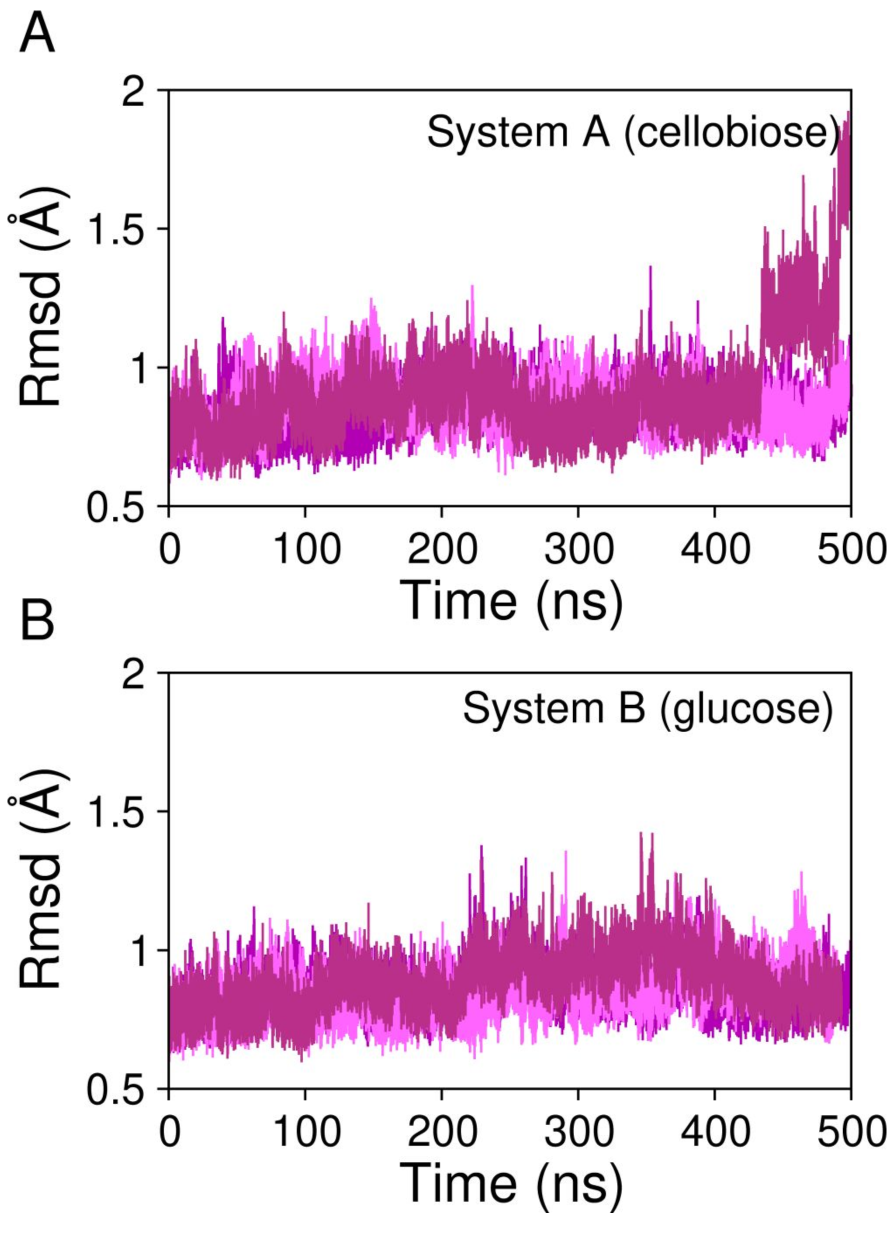

Figure S3. Root mean squared deviations (Rmsd) of the α-carbons of *Hi*Bgl relative to the crystal structure. (A) System A, *Hi*Bgl+cellobiose. (B) System B, *Hi*Bgl+glucose. Different colors mean different independent simulations.

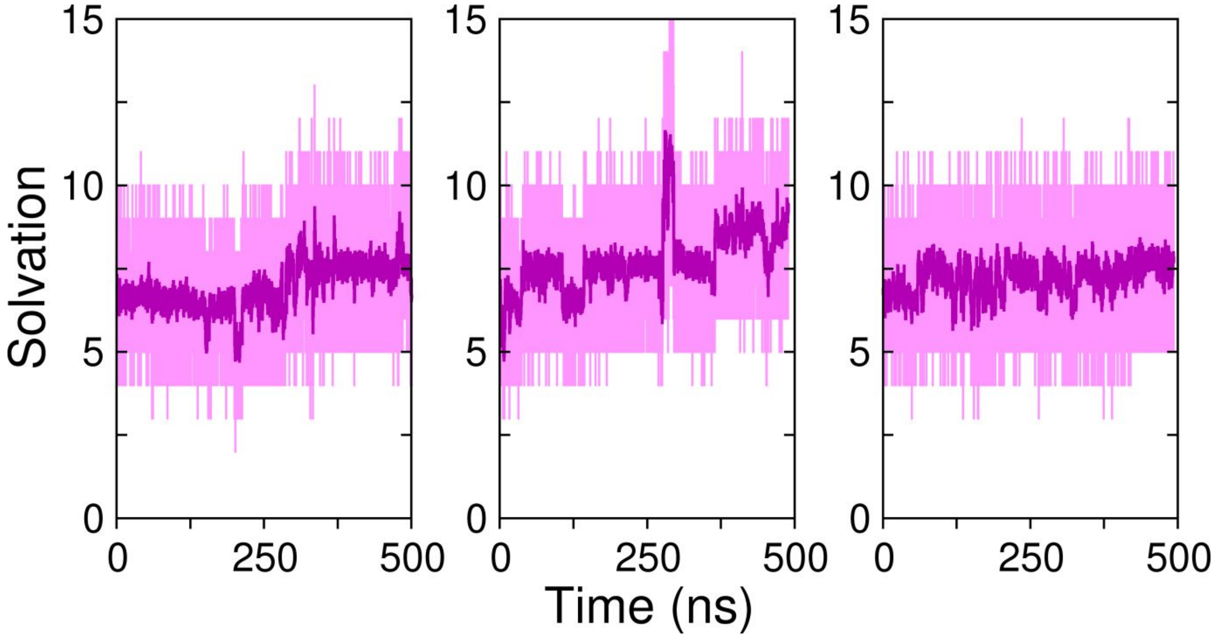

Figure S4. Solvation – defined as the number of water molecules within a shell of 3 Å – of glucose in the *Hi*Bgl–glucose complex (system B) in three independent MD simulations. The dark-colored curves represent moving averages of the solvation number, represented by the light-colored curves.

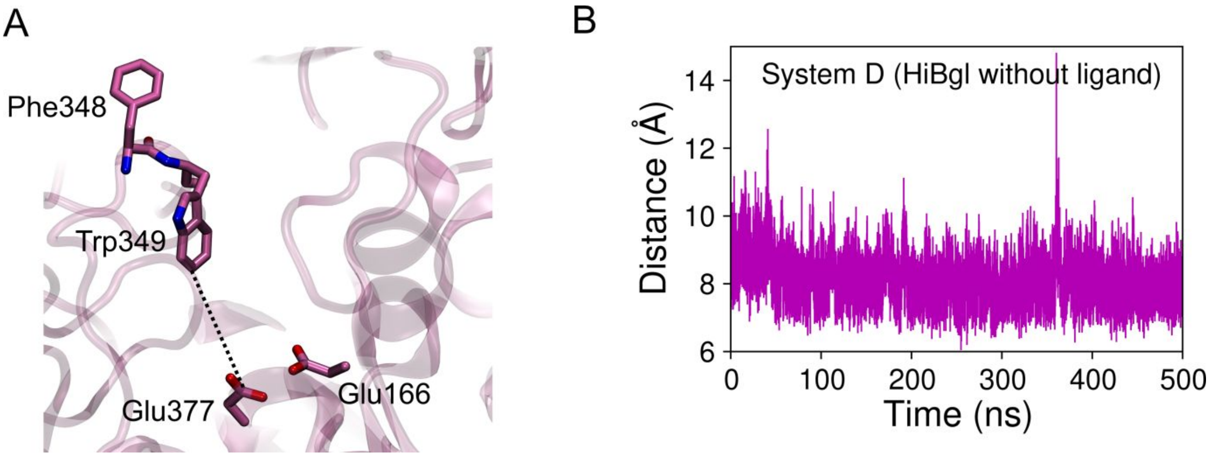

Figure S5. (A) Snapshot of *Hi*Bgl without any ligand (system D) showing residue Trp349 as part of subsite +1. (B) Distance between the CD atom of the catalytic base Glu377 and the CH2 atom of Trp349 – represented as a dotted line in panel A. This distance fluctuates around ~8 Å, which represents the conformation shown in panel A.

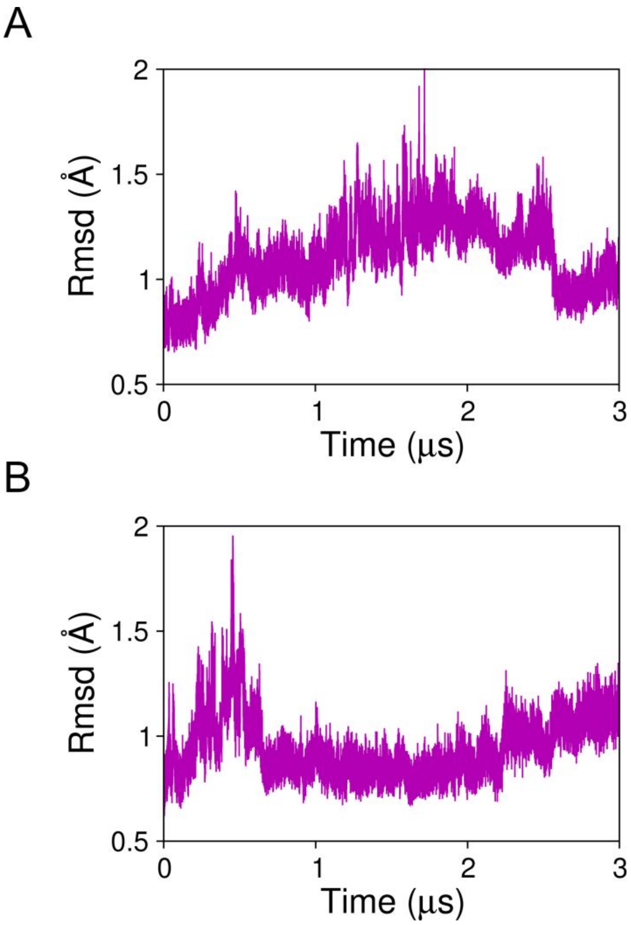

Figure S6. Root mean squared deviations (rmsd) of the α-carbons of *Hi*Bgl relative to the crystal structure in systems (A) F and (B) G.

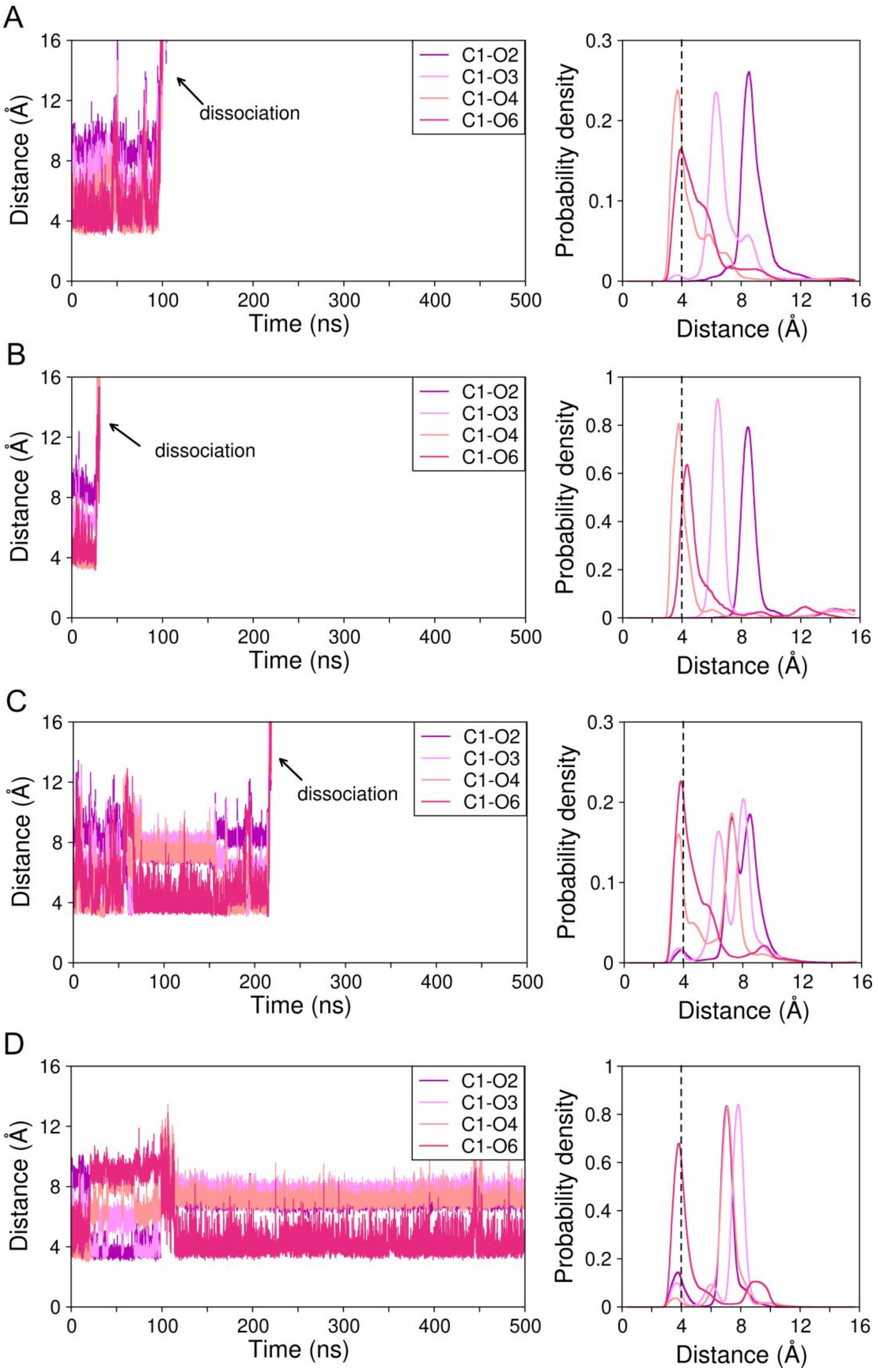

Figure S7. Distance between the C1 atom of glucose bound to subsite –1 and the O2, O3, O4, and O6 atoms of the glucose bound to subsite +1 along the simulation in which it dissociates in (A) ~100 ns, (B) ~30 ns, (C) ~200 ns, and (D) along the simulation in which it does not dissociate in 500 ns. On the right panels, the probability density distributions of the distances shown on the left panels are presented. In all cases, we can see that glucose at subsite +1 can assume different orientations relative to glucose at subsite –1.
